# Validation and functional characterization of GWAS-identified variants for chronic lymphocytic leukemia: a CRuCIAL study

**DOI:** 10.1038/s41408-022-00676-8

**Published:** 2022-05-17

**Authors:** Paloma García-Martín, Ana Moñiz Díez, José Manuel Sánchez Maldonado, Antonio José Cabrera Serrano, Rob ter Horst, Yolanda Benavente, Stefano Landi, Angelica Macauda, Alyssa Clay-Gilmour, Francisca Hernández-Mohedo, Yasmeen Niazi, Pedro González-Sierra, Blanca Espinet, Juan José Rodríguez-Sevilla, Rossana Maffei, Gonzalo Blanco, Matteo Giaccherini, Anna Puiggros, James Cerhan, Roberto Marasca, Marisa Cañadas-Garre, Miguel Ángel López-Nevot, Tzu Chen-Liang, Hauke Thomsen, Irene Gámez, Víctor Moreno, Rafael Marcos-Gragera, María García-Álvarez, Javier Llorca, Andrés Jerez, Sonja Berndt, Aleksandra Butrym, Aaron D. Norman, Delphine Casabonne, Mario Luppi, Susan L. Slager, Kari Hemminki, Yang Li, Miguel Alcoceba, Daniele Campa, Federico Canzian, Silvia de Sanjosé, Asta Försti, Mihai G. Netea, Manuel Jurado, Juan Sainz

**Affiliations:** 1 Hospital Campus de la Salud, PTS Granada, Granada, Spain; 2 Genomic Oncology Area, GENYO, Centre for Genomics and Oncological Research: Pfizer/University of Granada/Andalusian Regional Government, PTS, Granada, Spain; 3 Hematology department, Virgen de las Nieves University Hospital, Granada, Spain; 4 Instituto de Investigación Biosanitaria IBs.Granada, Granada, Spain; 5 Department of Internal Medicine and Radboud Center for Infectious Diseases, Radboud University Medical Center, Nijmegen, The Netherlands; 6 CeMM Research Center for Molecular Medicine of the Austrian Academy of Sciences, Vienna, Austria; 7 Catalan Institute of Oncology, Bellvitge Biomedical Research Institute (IDIBELL), Consortium for Biomedical Research in Epidemiology and Public Health (CIBERESP) and University of Barcelona, Barcelona, Spain; 8 CIBER Epidemiología y Salud Pública (CIBERESP), Madrid, Spain; 9 Department of Biology, University of Pisa, Pisa, Italy; 10 Genomic Epidemiology Group, German Cancer Research Center (DKFZ), Heidelberg, Germany; 11 Department of Epidemiology & Biostatistics, Arnold School of Public Health, University of South Carolina, Greenville, SC USA; 12 Division of Pediatric Neurooncology, German Cancer Research Center (DKFZ), German Cancer Consortium (DKTK), Heidelberg, Germany; 13 Hopp Children’s Cancer Center (KiTZ), Heidelberg, Germany; 14 Molecular Cytogenetics Laboratory, Pathology Department, Hospital del Mar, Barcelona, Spain; 15 Translational Research on Hematological Neoplasms Group, Cancer Research Program, Institut Hospital del Mar d’Investigacions Mèdiques (IMIM), Barcelona, Spain; 16 Hematology Department, Hospital del Mar, Barcelona, Spain; 17 Department of Medical and Surgical Sciences, University of Modena and Reggio Emilia, AOU Policlinico, Modena, Italy; 18 Department of Quantitative Health Sciences, Mayo Clinic, Rochester, MN 55905 USA; 19 Immunology Department, Virgen de las Nieves University Hospital, Granada, Spain; 20 Hematology Department, Morales Meseguer University Hospital, Murcia, Spain; 21 ProtaGene CGT GmbH, Heidelberg, Germany; 22 Unit of Biomarkers and Susceptibility, Cancer Prevention and Control Program, IDIBELL, Catalan Institute of Oncology; Department of Clinical Sciences, Faculty of Medicine, University of Barcelona, Barcelona, Spain; 23 Epidemiology Unit and Girona Cancer Registry, Oncology Coordination Plan, Department of Health, Autonomous Government of Catalonia, Catalan Institute of Oncology, Girona Biomedical Research Institute (IdiBGi), and Universitat de Girona, Girona, Spain; 24 Josep Carreras Leukemia Research Institute, Girona, Spain; 25 Department of Hematology, University Hospital of Salamanca (HUS/IBSAL), CIBERONC and Cancer Research Institute of Salamanca-IBMCC (USAL-CSIC), Salamanca, Spain; 26 University of Cantabria, Santander, Spain; 27 Division of Cancer Epidemiology and Genetics, National Cancer Institute, National Institutes of Health, Bethesda, MD USA; 28 Medical University of Wrocław, Wrocław, Poland; 29 Division of Computational Genomics, Mayo Clinic, Rochester, MN USA; 30 Division of Hematology, Mayo Clinic, Rochester, MN USA; 31 Division of Cancer Epidemiology, German Cancer Research Center (DKFZ), Im Neuenheimer Feld 280, 69120 Heidelberg, Germany; 32 Faculty of Medicine and Biomedical Center in Pilsen, Charles University in Prague, 30605 Pilsen, Czech Republic; 33 Centre for Individualised Infection Medicine (CiiM) & TWINCORE, joint ventures between the Helmholtz-Centre for Infection Research (HZI) and the Hannover Medical School (MHH), Hannover, Germany; 34 Department for Immunology & Metabolism, Life and Medical Sciences Institute (LIMES), University of Bonn, 53115 Bonn, Germany; 35 Department of Biochemistry and Molecular Biology I, Faculty of Sciences, University of Granada (UGR), Granada, Spain

**Keywords:** Risk factors, Genetics research

**Dear Editor,**

During the past years, considerable efforts have been made to uncover the genetic component of chronic lymphocytic leukemia (CLL) susceptibility. To date, several genome-wide association studies (GWAS) and their meta-analysis have identified not only single-nucleotide polymorphisms (SNPs) associated with CLL risk [1] but also patient survival [2]. However, despite these noticeable results, it becomes evident that both validation and functional characterization of the genetic variations identified are still required before they can be used in a clinical setting. Hence, we decided to validate the association of 41 GWAS-identified hits for CLL in 1158 CLL cases and 1947 controls ascertained through the Consortium for Research in Chronic lymphocytIc Leukemia (CRuCIAL) and to investigate their impact on modulating host immune responses and their utility to predict disease onset. Study participants were of European ancestry and gave their written informed consent to participate in the study, which was approved by the ethical review committee of participant institutions. CLL patients had often Binet stage A and Rai stage I (67.00% and 79.83%) and, compared to controls, had a higher mean age (66.19 ± 12.66 vs. 55.60 ± 11.50) and an increased male/female ratio (1.54 vs. 0.91). SNPs selection was based on published GWAS, functionality according to *HaploReg* data, and linkage disequilibrium between the SNPs. Genotyping of genetic variants was performed using KASP^TM^ and Taqman® assays. Hardy–Weinberg equilibrium was assessed in the controls (*P* > 0.001) and the association between CLL and SNPs was tested using a multivariate unconditional logistic regression analysis adjusted for age, sex, and country of origin. A meta-analysis of the CRuCIAL results with those from previous GWAS was conducted to validate genetic associations and the I [2] statistic was used to assess statistical heterogeneity between the studies (*P*_Het_ > 0.01). The pooled OR was computed using the fixed-effect model and the significance threshold for the meta-analysis was set to 5.0 × 10^−8^. Mechanistically, we evaluated the correlation of the GWAS-identified SNPs with a production of nine cytokines after in vitro stimulation of whole blood, peripheral mononuclear cells, and monocyte-derived macrophages from 408 healthy subjects of the Human Functional Genomic Project (HFGP) with LPS, PHA, Pam3Cys, CpG and *Borrelia burgdorferi* and *Escherichia coli*. In parallel, we also tested the correlation between selected SNPs and circulating concentrations of 103 serum and plasmatic inflammatory proteins, 7 plasma steroid hormones, and absolute numbers of 91 blood-derived immune cell populations. The HFGP study was approved by the Arnhem-Nijmegen Ethical Committee (42561.091.12) and biological specimens were collected after informed consent was obtained. A detailed description of the study population and participating centers, selected SNPs and protocols and reagents used in the functional experiments are included in the Supplementary Material available on the *Blood Cancer Journal* website. In order to account for multiple comparisons, we used a significance threshold of 2.3 × 10^−5^, 1.2 × 10^−5^, 1.34 × 10^−5^, and 1.74 × 10^−4^ for the cytokine quantitative trait loci, proteomic, blood cell counts, and steroid hormone analyses, respectively.

Logistic regression analyses confirmed the association of 21 SNPs with CLL risk at *P* < 0.05 level in the CRuCIAL cohort. The strongest association was found for SNPs located in the *GRAMD1B* locus (*P* = 6.2 × 10^−16^ and 6.0 × 10^−4^) that was further validated through meta-analysis (Table 1). The *GRAMD1B* locus (11q24.1) encodes for a transporter mediating the non-vesicular transport of cholesterol from the plasma membrane to the endoplasmic reticulum. Our experiments revealed that carriers of the *GRAMD1B*_rs35923643G_ allele had increased numbers of transitional CD24^+^CD38^+^ B cells (*P* = 4.25 × 10^−5^; Fig. 1A), which have an IL10-dependent immunosuppressive effect on pro-inflammatory responses against cancer cells. We also found that carriers of the *GRAMD1B*_rs35923643G_ allele had increased serum concentrations of IL18R1 (*P* = 0.00085; Fig. 1B), a receptor found to be dysregulated in CLL and that contributes to tumor escape from the immune system [3]. In support of the association of the *GRAMD1B*_rs35923643_ SNP with CLL risk, we found that this genetic variant is located among histone marks for primary B cells and it determines altered motifs for PU1, MEF2A, POU2F2, NKFB, OCT2 and IRF4, which is linked to CLL onset [1]. Moreover, we observed that carriers of the *GRAMD1B*_rs2953196G_ allele had decreased circulating concentrations of SIRT2 and ADA (*P* = 0.00037 and 0.00079; Fig. 1C, D). SIRT2 is overexpressed in primary CLL cells and plays a key role in determining cell survival [4]. Recent studies have shown that increased serum levels of SIRT2 were associated with longer overall survival [5] whereas SIRT2 inhibitors induced cell death in leukemic cell lines [6]. Similarly, ADA, an enzyme of the purine metabolism related to lymphoid T cell differentiation and tumor cellular responses, has been found to be overexpressed in CLL patients and correlates with longer survival [7]. Another study showed that blockade of A2A adenosine receptors made CLL cells more susceptible to pharmacological treatments while restoring immune competence and T cell proliferation [8]. Serra and coworkers also showed that activation of the ADO receptors inhibited chemotaxis and limited drug-induced apoptosis of CLL cells [9]. Finally, we found that carriers of the *GRAMD1B*_rs2953196G_ allele had decreased serum concentrations of STAMBP protein (*P* = 0.00033; Fig. 1E), a key protein involved in the control of autophagy flux and the NLRP3 inflammasome. These results suggest that the *GRAMD1B* locus might exert its biological function on CLL by modulating SIRT2, STAMBP, and ADA, which is a diagnostic biomarker for CLL that has been included in a new prognosis score designed to optimize the patient risk stratification [7].

**Table 1:** Validation of GWAS-identified variants for CLL.

SNP	Chr.	Nearby gene	Risk allele	CRuCIAL consortium (*n* = 3105; 1158 CLL cases and 1947 controls)		Previously published GWAS^b^		Meta-analysis		
				OR (95% CI)^a^	*P*	OR (95% CI)	*P*	OR (95% CI)	*P*	*P*_Het_
rs4368253	18	*AC107990.1*||*NFE2L3P1*	C	**1.30 (1.13–1.49)**	**2.0E−04**	**1.18 (1.11–1.26)**	**8.00E−07**	**1.20 (1.13–1.27)**	**5.55E−10**	0.212
rs58055674	2	*ACOXL*	C	**1.17 (1.01–1.35)**	**0.032**	**1.44 (1.33–1.56)**	**5.00E−20**	**1.37 (1.28–1.47)**	**7.01E−19**	0.014
rs1439287	2	ACOXL	T	**1.26 (1.11–1.41)**	**0.0002**	**1.37 (1.26–1.47)**	**5.00E−15**	**1.34 (1.25–1.43)**	**1.62E−18**	0.249
rs7944004	11	*ASCL2*||*C11orf21*	T	1.10 (0.97–1.24)	0.12	**1.20 (1.13–1.27)**	**2.00E−10**	**1.18 (1.12–1.25)**	**6.44E−10**	0.209
rs4987855	18	*BCL2*	G	**1.42 (1.15–1.76)**	**0.0012**	**1.47 (1.32–1.61)**	**3.00E−12**	**1.46 (1.34–1.60)**	**1.50E−16**	0.773
rs2651823	11	*C11orf21*|*TSPAN32*	A	1.09 (0.97–1.23)	0.15	**1.18 (1.13–1.25)**	**5.00E−11**	**1.17 (1.11–1.22)**	**9.62E−11**	0.228
rs1476569	4	*CAMK2D*	G	0.97 (0.86–1.10)	0.66	**1.18 (1.12–1.25)**	**6.00E−10**	1.14 (1.09–1.20)	2.01E−07	**0.004**
rs3769825	2	*CASP8*	T	1.05 (0.93–1.19)	0.40	***1.19 (1.12–1.25)***	**3.00E−09**	**1.17 (1.11–1.23)**	**2.12E−09**	0.069
rs7558911	2	*CFLAR*	A	1.02 (0.91–1.16)	0.71	**1.18 (1.12–1.24)**	**5.00E−11**	**1.16 (1.10–1.21)**	**1.94E−09**	0.030
rs1036935	18	*CXXC1*	A	**1.20 (1.05–1.38)**	**0.0091**	**1.15 (1.10–1.21)**	**3.00E−08**	**1.16 (1.10–1.21)**	**3.02E−10**	0.564
rs1359742	9	*DMRTA1*	G	**1.18 (1.04–1.32)**	**0.0078**	**1.20 (1.12–1.28)**	**7.00E−09**	**1.20 (1.13–1.27)**	**1.98E−09**	0.809
rs6546149	2	*DTNB*	G	1.05 (0.92–1.20)	0.46	1.09 (1.01–1.17)	2.14E−02	1.08 (1.01–1.15)	0.018	0.629
rs9880772	3	*EOMES*|*LINC01980*	T	**1.27 (1.13–1.43)**	**5.97E−05**	**1.19 (1.13–1.25)**	**2.55E−11**	**1.20 (1.15–1.26)**	**7.39E−15**	0.319
rs13015798	2	*FAM126B*	A	0.98 (0.86–1.13)	0.82	**1.20 (1.14–1.30)**	**3.00E−08**	1.15 (1.09–1.23)	1.76E−06	**0.009**
rs6586163	10	*FAS*	A	**1.29 (1.14–1.46)**	**4.50E−05**	**1.23 (1.17–1.29)**	**1.00E−15**	**1.24 (1.18–1.30)**	**3.20E−20**	0.483
rs2267708	7	*GPR37*	T	**1.22 (1.08–1.37)**	**0.0012**	**1.16 (1.10–1.22)**	**9.00E−09**	**1.17 (1.12-1.23)**	**1.04E−10**	0.446
rs35923643	11	*GRAMD1B*	G	**1.93 (1.66–2.24)**	**6.20E−16**	**1.66 (1.54-1.79)**	**2.00E−40**	**1.71 (1.60–1.83)**	**2.76E−55**	0.078
rs2953196	11	*GRAMD1B*	G	**1.29 (1.12–1.49)**	**6.00E−04**	**1.30 (1.22–1.38)**	**5.00E−16**	**1.30 (1.23–1.37)**	**1.45E−19**	0.922
rs3800461	6	*ILRUN*	C	1.12 (0.90–1.38)	0.32	**1.20 (1.13–1.28)**	**1.97E−08**	**1.19 (1.12–1.27)**	**6.80E−09**	0.544
rs9392504	6	*IRF4*	A	**1.46 (1.29–1.64)**	**1.75E−09**	**1.33 (1.26–1.40)**	**1.00E−28**	**1.35 (1.29–1.42)**	**3.14E−34**	0.163
rs391855	16	*IRF8*	A	**1.20 (1.06–1.36)**	**0.0031**	**1.37 (1.28–1.45)**	**1.00E−22**	**1.33 (1.26–1.41)**	**3.94E−24**	0.062
rs898518	4	*LEF1*	A	0.95 (0.84–1.08)	0.41	**1.20 (1.14–1.27)**	**4.00E−10**	**1.15 (1.10–1.21)**	**1.16E−08**	**0.001**
rs34676223	1	*MDS2*	C	**1.17 (1.03–1.33)**	**0.015**	**1.19 (1.14–1.25)**	**5.04E−13**	**1.19 (1.14–1.24)**	**7.23E−15**	0.807
rs57214277	4	*MYL12BP2*||*LINC02363*	T	1.06 (0.92–1.23)	0.43	**1.13 (1.08–1.18)**	**3.69E−08**	1.12 (1.08–1.17)	6.50E−08	0.409
rs10936599	3	*MYNN*	C	1.12 (0.96–1.29)	0.14	**1.26 (1.17–1.35)**	**1.74E−09**	**1.22 (1.16–1.31)**	**2.11E−10**	0.160
rs11715604	3	NCK1	T	0.98 (0.84–1.14)	0.80	NA (NA-NA)	**1.97E−08**	NA (NA-NA)	NA	NA
rs6489882	12	*OAS3*	G	1.09 (0.96–1.23)	0.19	**1.16 (1.10–1.22)**	**5.00E−08**	**1.15 (1.10–1.21)**	**1.13E−08**	0.364
rs140522	22	*ODF3B*	T	**1.17 (1.03–1.32)**	**0.016**	**1.15 (1.10–1.20)**	**2.70E−09**	**1.15 (1.11–1.20)**	**1.35E−11**	0.797
rs2236256	6	*OPRM1*||*IPCEF1*	C	**1.20 (1.06–1.35)**	**0.0037**	**1.23 (1.15–1.30)**	***1.50E−10***	**1.22 (1.16–1.29)**	**4.49E−13**	0.721
rs11637565	15	PCAT29|LOC107984788	G	**1.20 (1.06–1.36)**	**0.0040**	**1.35 (1.28–1.42)**	***2.00E−31***	**1.33 (1.27–1.39)**	**6.19E−31**	0.087
rs17246404	7	*POT1*	C	**1.28 (1.12–1.46)**	**3.68E−04**	**1.22 (1.14–1.31)**	**3.40E−08**	**1.23 (1.16–1.31)**	**2.71E−11**	0.530
rs2511714	8	*POU5F1P2*||*ODF1*	G	1.08 (0.95–1.22)	0.23	**1.19 (1.11–1.27)**	**2.00E−07**	1.16 (1.10–1.24)	4.89E−07	0.181
rs11083846	19	*PRKD2*	A	**1.17 (1.01–1.33)**	**0.030**	**1.35 (1.22–1.49)**	**3.96E−09**	**1.29 (1.19–1.39)**	**1.24E−09**	0.099
rs888096	2	*QPCT*||*RNU6-1116P*	A	1.09 (0.97–1.24)	0.16	**1.15 (1.09–1.21)**	**5.00E−08**	**1.14 (1.09–1.20)**	**5.37E−08**	0.431
rs41271473	1	*RHOU*	G	0.95 (0.80–1.12)	0.52	**1.19 (1.13–1.26)**	**1.06E−10**	**1.17 (1.11–1.23)**	**7.76E−09**	0.013
rs73718779	6	*SERPINB6*	A	0.93 (0.76–1.14)	0.47	**1.26 (1.16–1.36)**	**1.97E−08**	1.21 (1.12**–**1.30)	4.51E−07	**0.006**
rs12638862	3	TERC	A	1.09 (0.94–1.25)	0.25	**1.15 (1.09–1.19)**	**2.00E−11**	**1.15 (1.10–1.19)**	**2.72E−10**	0.481
rs7705526	5	TERT	A	**1.27 (1.11–1.45)**	**5.0E−04**	**1.18 (1.12–1.25)**	**6.00E−10**	**1.19 (1.13–1.26)**	**1.06E−11**	0.319
rs61904987	11	*TMPRSS5*||*DRD2*	T	**1.24 (1.02–1.52)**	**0.032**	**1.24 (1.16–1.32)**	**2.46E−11**	**1.24 (1.17–1.32)**	**6.90E−12**	1.000
rs926070	6	TSBP1-AS1	A	1.08 (0.95–1.24)	0.24	**1.27 (NA–NA)**	**4.00E−08**	NA (NA-NA)	NA	NA
rs7254272	19	*ZBTB7A*|*MAP2K2*	A	1.06 (0.91–1.22)	0.46	**1.17 (1.10–1.23)**	**4.67E−08**	1.15 (1.10–1.21)	5.93E−08	0.202

OR and 95% CI was not reported in the original study and, therefore, association estimates were not available in the GWAS catalog. In bold in the CRuCIAL cohort, SNPs with *P* values <0.05. In bold in the meta-analyses, SNPs with a *P* value <5.5 × 10^−8^ (gold standard significance threshold for GWAS). Considering the relatively low power of the CRuCIAL cohort, *P*_Het_ threshold was set to 0.01.

*NA* not available.

^a^Association estimates calculated according to a log-additive model of inheritance and adjusted for age, sex and country of origin.

^b^References (Pubmed ID) to previously published GWAS are included in the Supplementary Material available on the *Blood Cancer Journal* website.

**Fig. 1:**
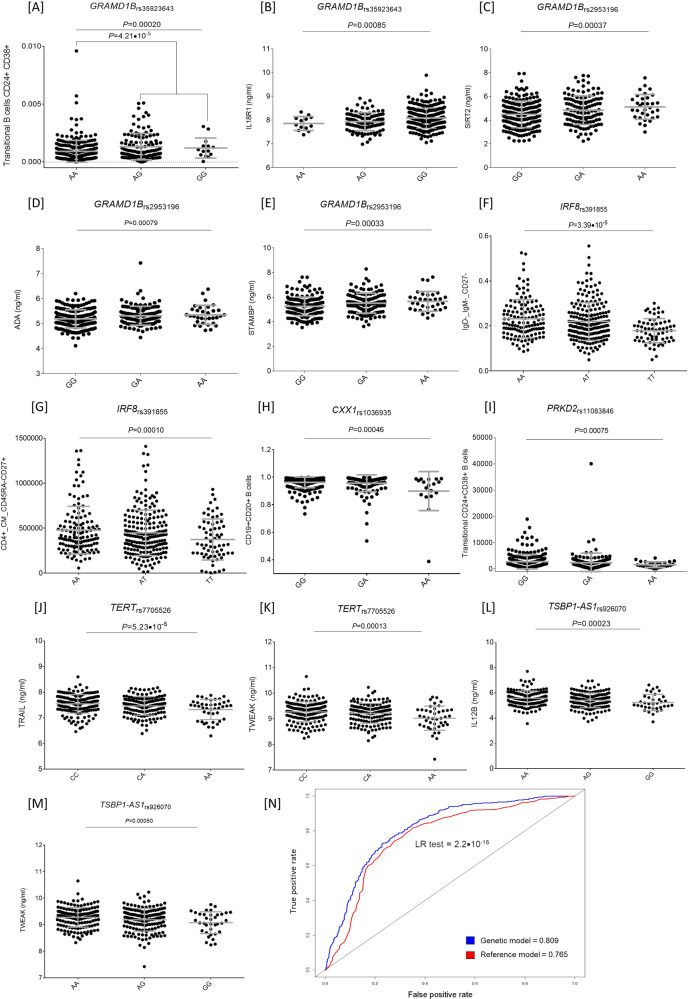
Functional characterization of GWAS-identified variants for CLL (A–M) and receiver operating characteristic (ROC) curve analysis (N). Correlation between functional data and GWAS-identified SNPs was evaluated by linear regression analysis adjusted for age and sex. ROC curve summarizes the accuracy of prediction for each particular model. The model including SNPs significantly associated with the risk of developing CLL and demographic variables (marked in blue) showed a significantly improved predictive capacity compared with a reference model including only age and gender as covariates (marked in red). AUC = 0.809 vs. AUC = 0.765; *N* = 2123 subjects; LR test = 2.2 × 10^−16^.

Besides these findings, the meta-analysis confirmed the association of 29 additional SNPs with the risk of developing the disease (OR_Meta_ = 1.15–1.71; Table 1), which suggested a functional role of these markers in modulating CLL risk. In this regard, our experiments revealed that carriers of the *IRF8*_rs391855A_ allele showed increased numbers of class-switched CD27^-^IgM^−^IgD^−^ memory B cells (*P* = 3.39 × 10^−5^; Fig. 1F) and central memory CD4^+^CD45RA^−^CD27^+^ T cells (*P* = 0.0001; Fig. 1G), whereas carriers of the *CXXC1*_rs1036935A_ allele had decreased numbers of CD19^+^CD20^+^ B cells (*P* = 0.00075; Fig. 1H), a subset of cells poorly expressed in CLL patients [10]. The *IRF8* locus encodes for a transcription factor exclusively expressed in immune cells that regulate B cell-activating factor (BAFF)-mediated B cell activation, cell survival, adaptative NK cell responses, and CD8/CD4 T cell differentiation. In line with these findings, we also found that carriers of the *PRKD2*_rs11083846A_ allele showed decreased numbers of transitional CD24+ CD38+ B cells (*P* = 0.00046; Fig. 1I), whereas carriers of the *ILRUN*_rs3800461C_ allele had decreased levels of HLA-DR^+^ T regulatory and conventional CD4^+^ T cells. Finally, we also observed that carriers of the *POU5F1P2*_rs2511714G_ allele showed increased numbers of CD8^+^ effector memory (CD45RA^−^CD27^−^) T cells. The *POU5F1P2*_rs2511714G_ SNP is located among histone marks in primary B cells whereas the *PRKD2*_rs11083846_ SNP is an eQTL for the *PRKD2* gene in whole blood but also *SLC1A5*, *CALM3*, and *FKRP* genes that have been associated with CLL onset [11]. We hypothesize that the *IRF8*, *CXX1*, *ILRUN*, and *POU5F1P*|*ODF1* loci might influence CLL risk by modulating specific subsets of B and T cells and regulatory T cells that play critical roles in the pathogenesis of the disease [12] and influence prognosis. In fact, it is known that peripheral regulatory T cell populations expressing CD4^+^ in CLL are associated with disease progression and exhibit a prognostic value [13]. In addition, we found a correlation between the *TERT*_rs7705526A_ allele and decreased serum concentrations of TRAIL and TWEAK (*P* = 5.23 × 10^−5^ and 0.0001; Fig. 1J, K), which are involved in the regulation of key cell functions including immune responses, inflammation, proliferation, differentiation, and apoptosis. These results are in agreement with those showing that CLL patients exhibit reduced serum TRAIL both before and after treatment [14] and that its aberrant expression in CLL promotes cell survival [15]. Similarly, we found a correlation of the *TSBP1-AS1*_rs926070G_ allele with decreased concentrations of IL12 and TWEAK proteins (*P* = 0.00023 and 0.00050; Fig. 1L, M), which reinforced the idea of an implication of TWEAK and TWEAK-mediated immune responses in CLL. In support of this finding, it has been reported that TWEAK attenuates the transition from innate to adaptive immunity, which might affect blood cell populations, immune responses, and, consequently, influence the susceptibility to CLL. On the other hand, we found that carriers of the *ILRUN*_rs3800461C_ allele had decreased numbers of conventional CD4+ T cells and HLA-DR+ T regulatory cells (*P* = 0.00041 and 0.00058), whereas carriers of the *POU5F1P2*_rs2511714G_ allele showed increased numbers of CD8+ effector memory CD45RA-CD27- cells (*P* = 0.00053; Supplementary Material). No functional effect for the rest of SNPs was observed.

Considering the number of variants that showed significant associations with CLL risk, we attempted to establish the clinical usefulness of genetic biomarkers in predicting disease onset by using a double approach that consisted of building a predictive model using demographic variables and SNPs significantly associated with CLL risk and weighted and unweighted polygenic risk scores (PRSs; Supplementary Material). The area under the curve (AUC) of a receiver operating characteristic curve analysis and −2 log-likelihood ratio (LR) tests showed that a model including age, sex, and 16 SNPs significantly improved the ability to predict the onset of the disease when compared with the reference model including only demographic variables (AUC = 0.809 vs AUC = 0.765; *P*_LRtest_ = 2.2 × 10^−16^; Fig. 1N). We also computed weighted and unweighted PRSs in a subset of 806 CLL cases and 1417 controls from the CRuCIAL cohort and we found an OR = 6.81, 95% CI 4.65–9.96, *P* = 2.0 × 10^−21^ for the highest vs. lowest quintile of the unweighted score and OR = 10.45, 95% CI 6.96–15.70, *P* = 2.0 × 10^−27^ for the highest vs. lowest quintile of the weighted score. Strong associations were also observed when weighted scores were built using ORs from the original GWASs. The best AUC value was observed for the weighted score computed in the CRuCIAL cohort (AUC = 0.68, 95% CI 0.65–0.70).

In conclusion, this study confirmed the association of 31 GWAS-identified SNPs with CLL risk and shed some light on the function of some of these biomarkers in the modulation of T_Reg_, B, and T cell differentiation and proliferation, blood concentrations of B cell-related proteins, cell survival, and the expression of immune- and non-immune-related loci. Though outside the scope of the current study, it is important to mention that additional functional studies using blood samples from CLL patients are still required to validate our findings and to decipher the exact biological mechanisms behind the observed associations. A potential limitation of this work was the relatively small population size of the CRuCIAL cohort that hampered the validation of the SNPs showing modest associations.

## Supplementary information

Supplementary Material

## Data Availability

The genotype data used in the present study are available by the corresponding authors upon reasonable request. Functional data used in this project have been meticulously cataloged and archived in the BBMRI-NL data infrastructure (https://hfgp.bbmri.nl/) using the MOLGENIS open-source platform for scientific data. This allows flexible data querying and download, including sufficiently rich metadata and interfaces for machine processing (R statistics, REST API) and using FAIR principles to optimize Findability, Accessibility, Interoperability, and Reusability.

## References

[1] Berndt SI, Camp NJ, Skibola CF, Vijai J, Wang Z, Gu J (2016). Meta-analysis of genome-wide association studies discovers multiple loci for chronic lymphocytic leukemia. Nat Commun.

[2] Lin WY, Fordham SE, Sunter N, Elstob C, Rahman T, Willmore E (2021). Genome-wide association study identifies risk loci for progressive chronic lymphocytic leukemia. Nat Commun.

[3] Airoldi I, Raffaghello L, Cocco C, Guglielmino R, Roncella S, Fedeli F (2004). Heterogeneous expression of interleukin-18 and its receptor in B-cell lymphoproliferative disorders deriving from naive, germinal center, and memory B lymphocytes. Clin Cancer Res.

[4] Bhalla S, Gordon LI (2016). Functional characterization of NAD dependent de-acetylases SIRT1 and SIRT2 in B-cell chronic lymphocytic leukemia (CLL). Cancer Biol Ther.

[5] Van Damme M, Crompot E, Meuleman N, Mineur P, Bron D, Lagneaux L (2012). HDAC isoenzyme expression is deregulated in chronic lymphocytic leukemia B-cells and has a complex prognostic significance. Epigenetics.

[6] Dan L, Klimenkova O, Klimiankou M, Klusman JH, van den Heuvel-Eibrink MM, Reinhardt D (2012). The role of sirtuin 2 activation by nicotinamide phosphoribosyltransferase in the aberrant proliferation and survival of myeloid leukemia cells. Haematologica.

[7] Yun X, Sun X, Hu X, Zhang H, Yin Z, Zhang X (2021). Prognostic and therapeutic value of apolipoprotein A and a new risk scoring system based on apolipoprotein a and adenosine deaminase in chronic lymphocytic leukemia. Front Oncol.

[8] Serra S, Vaisitti T, Audrito V, Bologna C, Buonincontri R, Chen SS (2016). Adenosine signaling mediates hypoxic responses in the chronic lymphocytic leukemia microenvironment. Blood Adv.

[9] Serra S, Horenstein AL, Vaisitti T, Brusa D, Rossi D, Laurenti L (2011). CD73-generated extracellular adenosine in chronic lymphocytic leukemia creates local conditions counteracting drug-induced cell death. Blood.

[10] Ginaldi L, De Martinis M, Matutes E, Farahat N, Morilla R, Catovsky D (1998). Levels of expression of CD19 and CD20 in chronic B cell leukaemias. J Clin Pathol.

[11] Sille FC, Thomas R, Smith MT, Conde L, Skibola CF (2012). Post-GWAS functional characterization of susceptibility variants for chronic lymphocytic leukemia. PLoS ONE.

[12] De Matteis S, Molinari C, Abbati G, Rossi T, Napolitano R, Ghetti M (2018). Immunosuppressive Treg cells acquire the phenotype of effector-T cells in chronic lymphocytic leukemia patients. J Transl Med.

[13] Mpakou VE, Ioannidou HD, Konsta E, Vikentiou M, Spathis A, Kontsioti F (2017). Quantitative and qualitative analysis of regulatory T cells in B cell chronic lymphocytic leukemia. Leuk Res.

[14] Jablonska E, Kiersnowska-Rogowska B, Aleksandrowicz-Bukin M, Rogowski F, Sawicka-Powierza J (2008). TRAIL receptors in the serum of patients with B-cell chronic lymphocytic leukemia. Neoplasma.

[15] Secchiero P, Tiribelli M, Barbarotto E, Celeghini C, Michelutti A, Masolini P (2005). Aberrant expression of TRAIL in B chronic lymphocytic leukemia (B-CLL) cells. J Cell Physiol.

