# Supplementary material for: Validation and functional characterization of GWAS-identified variants for chronic lymphocytic leukemia: a CRuCIAL study
Source: Blood Cancer J. 2022 May 17;12(5):79. doi: 10.1038/s41408-022-00676-8 (PMC9114372; doi:10.1038/s41408-022-00676-8)
Supplement: Supplementary file 1 — Supplementary Material [file 41408_2022_676_MOESM1_ESM.docx]

**SUPPLEMENTARY MATERIAL**

**MATERIAL AND METHODS**

*Study populations*

The population consisted of 1158 CLL patients and 1947 healthy controls ascertained through the Consortium for Research in Chronic lymphocytIc Leukemia (CRuCIAL). CLL patients fulfilled the revised diagnostic criteria^1,2^. Study participants were of European ancestry and gave their written informed consent to participate in the study, which was approved by the ethical review committee of participant institutions: Virgen de las Nieves University Hospital (Granada, Spain, 0760-N-18); University Hospital of Salamanca (Salamanca, Spain, PI90/07/2018); Hospital del Mar (Barcelona, Spain); Catalan Institute of Oncology (Barcelona, Spain); Morales Meseguer University Hospital (Murcia, Spain); Consortium for Biomedical Research in Epidemiology and Public Health (CIBERESP) group (Spain); University of Modena and Reggio Emilia, AOU Policlinico (Modena, Italy); University of Pisa (Pisa, Italy), Wroclaw Medical University (Wroclaw, Poland) and by the Radboud University Medical Center (Nijmegen, The Netherlands, 2011/299). A detailed description of the population and participating centers are shown in supplementary tables 1 and 2, respectively. The study followed the Declaration of Helsinki.

**Supplementary Table 1.** Characteristics of the CRuCIAL population.

| **CRuCIAL Population (n=3105)** | | | |
| --- | --- | --- | --- |
| **CLL (n=1158)** | | **Healthy controls (n=1947)** | |
| Age (years) | 66.19 ± 12.26 | 55.60 ± 11.50 |  |
| Sex ratio (male/female) | 1.54 (665/430) | 0.91 (882/966) |  |
| Country of origin |  |  |  |
| Spain | 780 | 1419 |  |
| Poland | 78 | 97 |  |
| Italy | 300 | 431 |  |
| Binet stage (%) |  |  |  |
| 0 | 149 (18.00) | - |  |
| A | 553 (66.78) | - |  |
| B | 90 (10.87) | - |  |
| C | 36 (4.34) | - |  |
| Rai Stage (%) |  |  |  |
| 0 | 27 (2.83) | - |  |
| I | 746 (78.20) | - |  |
| II | 125 (13.10) | - |  |
| III | 17 (1.78) | - |  |
| IV | 39 (4.09) | - |  |

Mean ± standard error

**Supplementary table 2.** List of participating centres.

| CRuCIAL cohort  Participant centers | Center (City) | CLL cases | Healthy controls |
| --- | --- | --- | --- |
| Spain | VNUH (Granada) | 121 | 1031 |
|  | ICO/IDIBELL (Barcelona) | 186 | 171 |
|  | HDM (Barcelona) | 114 | - |
|  | UHS (Salamanca) | 162 | 9 |
|  | MMUH (Murcia) | 197 | 211 |
| Italy |  |  |  |
|  | UMRE (Modena) | 300 | 431 |
| Poland |  |  |  |
|  | MUW (Wrocław) | 78 | 97 |

VNUH, Virgen de las Nieves University hospital; ICO, Catalan Institute of Oncology; HDM, Hospital del Mar; UHS, University Hopital of Salamanca; UMRE, University of Modena and Reggio Emilia; MUW, Medical University of Wrocław.

*DNA extraction, SNP selection, association analysis, and meta-analysis*

Genomic DNA from the CLL patients and the healthy controls was extracted from blood samples using the QIAamp DNA Blood Mini kit (Valencia, CA, EEUU) according to manufacturer’s instructions. Single nucleotide polymorphisms (SNPs) were selected through an extensive literature search of relevant GWAS and GWAS meta-analyses published by February 2019 using publicly available online databases^3-6^. The search terms used were: Genome-wide association analysis, risk, susceptibility, and chronic lymphocytic leukemia. Additional criteria were potential functionality according to *HaploReg* data and linkage disequilibrium between the SNPs. A total of 41 SNPs in 39 loci were selected for genotyping using KASP^TM^ (LGC Genomics, Hoddesdon, UK) and Taqman® assays (Thermo Fisher, USA; supplementary table 3). Hardy–Weinberg equilibrium was assessed in the control group (*P*>0.001) and the association between CLL and selected SNPs was tested using a multivariate unconditional logistic regression analysis adjusted for age, sex and country of origin. Overall statistical power in the CRuCIAL cohort was calculated using Quanto (v.12.4) assuming a log-additive model of inheritance and a baseline risk of 0.000047. Finally, we conducted a meta-analysis of the CRuCIAL results with those from a previous GWAS meta-analysis and the I^2^ statistic was used to assess statistical heterogeneity between the studies. The pooled OR was computed using the fixed effect model and the significance threshold for the meta-analysis was set to 5.0•10^-8^, as recommended for a GWAS.

**Supplementary table 3.** List of GWAS-identified variants for CLL.

| **SNP** | **Chr.** | **Nearby gene** | **Reference Allele** | **Risk allele** | **Location** | **Author (Pubmed ID)** |
| --- | --- | --- | --- | --- | --- | --- |
| rs4368253 | 18 | *AC107990.1\|\|NFE2L3P1* | T | C | Intergenic | Berndt SI (26956414) |
| rs58055674 | 2 | *ACOXL* | T | C | Intronic | Berndt SI (26956414) |
| rs1439287 | 2 | ACOXL | C | T | Intronic | Berndt SI (23770605) |
| rs7944004 | 11 | *ASCL2. C11orf21* | G | T | Intergenic | Berndt SI (23770605) |
| rs4987855 | 18 | *BCL2* | A | G | 3’ UTR | Berndt SI (23770605) |
| rs2651823 | 11 | *C11orf21\|TSPAN32* | G | A | Intronic | Law PJ (28165464) |
| rs1476569 | 4 | *CAMK2D* | A | G | Intergenic | Law PJ (28165464) |
| rs3769825 | 2 | *CASP8* | C | T | Intronic | Berndt SI (23770605) |
| rs7558911 | 2 | *CFLAR* | G | A | Intronic | Law PJ (28165464) |
| rs1036935 | 18 | *CXXC1* | G | A | Intergenic | Law PJ (28165464) |
| rs1359742 | 9 | *DMRTA1* | C | G | Intergenic | Berndt SI (26956414) |
| rs6546149 | 2 | *DTNB* | C | G | Intronic | Law PJ (28112199) |
| rs9880772 | 3 | *EOMES\|LINC01980* | C | T | Intergenic | Berndt SI (26956414) |
| rs13015798 | 2 | *FAM126B* | G | A | Intronic | Berndt SI (26956414) |
| rs6586163 | 10 | *FAS* | C | A | Intronic | Law PJ (28165464) |
| rs2267708 | 7 | *GPR37* | C | T | Intronic | Law PJ (28165464) |
| rs35923643 | 11 | *GRAMD1B* | A | G | Intronic | Berndt SI (26956414) |
| rs2953196 | 11 | *GRAMD1B* | A | G | Intronic | Berndt SI (26956414) |
| rs3800461 | 6 | *ILRUN* | G | C | Intronic | Law PJ (28165464) |
| rs9392504 | 6 | *IRF4* | G | A | Intergenic | Law PJ (28165464) |
| rs391855 | 16 | *IRF8* | T | A | Intergenic | Berndt SI (26956414) |
| rs898518 | 4 | *LEF1* | C | A | Intronic | Berndt SI (23770605) |
| rs34676223 | 1 | *MDS2* | A | C | Intergenic | Law PJ (28165464) |
| rs57214277 | 4 | *MYL12BP2\|\|LINC02363* | C | T | Intergenic | Law PJ (28165464) |
| rs10936599 | 3 | *MYNN* | T | C | Missense | Speedy HE (24292274) |
| rs11715604 | 3 | NCK1 | A | T | Intronic | Law PJ (28112199) |
| rs6489882 | 12 | *OAS3* | A | G | Intronic | Law PJ (28165464) |
| rs140522 | 22 | *ODF3B* | C | T | Upstream | Law PJ (28165464) |
| rs2236256 | 6 | *OPRM1\|\|IPCEF1* | A | C | Intronic/3’UTR | Speedy HE (24292274) |
| rs11637565 | 15 | PCAT29\|LOC107984788 | A | G | Intergenic | Law PJ (28165464) |
| rs17246404 | 7 | *POT1* | T | C | Non Coding Transcript Variant | Speedy HE (24292274) |
| rs2511714 | 8 | *POU5F1P2\|\|ODF1* | T | G | Intergenic | Berndt SI (26956414) |
| rs11083846 | 19 | *PRKD2* | G | A | Intronic | Di Bernardo MC (18758461) |
| rs888096 | 2 | *QPCT\|\|RNU6-1116P* | G | A | Intergenic | Law PJ (28165464) |
| rs41271473 | 1 | *RHOU* | A | G | Non Coding Transcript Variant | Law PJ (28165464) |
| rs73718779 | 6 | *SERPINB6* | G | A | Intronic | Berndt SI (26956414) |
| rs12638862 | 3 | TERC | G | A | Intergenic | Law PJ (28112199) |
| rs7705526 | 5 | TERT | C | A | Intronic | Law PJ (28165464) |
| rs61904987 | 11 | *TMPRSS5\|\|DRD2* | C | T | Intergenic | Law PJ (28165464) |
| rs926070 | 6 | TSBP1-AS1 | G | A | Intronic | Speedy HE (24292274) |
| rs7254272 | 19 | *ZBTB7A\|MAP2K2* | G | A | Intergenic | Law PJ (28165464) |

*Functional association of the GWAS-identified variants for CLL with immune responses*

In order to determine the functional role of the GWAS-identified SNPs after the meta-analysis of the CRuCIAL and GWAS cohorts, we conducted cytokine stimulation experiments in the 500 Functional Genomics cohort from the Human Functional Genomics Project (HFGP; <http://www.humanfunctionalgenomics.org/site/>). The HFGP study was approved by the Arnhem-Nijmegen Ethical Committee (no. 42561.091.12) and biological specimens were collected after informed consent was obtained. We investigated whether any of the 32 SNPs that were successfully validated through the meta-analysis of the study populations correlated with the levels of 9 pro- and anti-inflammatory cytokines (TNFα, IFNγ, IL1Ra, IL1β, IL6, IL8, IL10, IL17, and IL22) after the stimulation of whole blood, peripheral mononuclear cells (PBMCs) or macrophage-derived monocytes (MDM) from 408 healthy subjects with LPS (100ng/ml, Sigma-Aldrich, St. Louis), PHA (10μg/ml, Sigma), Pam3Cys (10μg/ml, EMC microcollections), CpG (100 ng/ml, InvivoGen), Bacteroides fragilis (NCTC 10584) and Staphylococcus Aureus (ATCC 25923) for 24 or 48 hours. Bacteroides fragilis and Staphylococcus Aureus were heat-killed for 30 min at 95ºC and 100ºC, respectively. Detailed protocols for PBMCs isolation, macrophage differentiation and stimulation assays have been reported elsewhere29. Briefly, PBMCs were washed twice in saline and suspended in medium (RPMI 1640) supplemented with gentamicin (10 mg/mL), L-glutamine (10 mM) and pyruvate (10 mM). PBMC stimulations were performed with 5•10^5^ cells/well in round-bottom 96-wells plates (Greiner) for 24 hours in the presence of 10% human pool serum at 37°C and 5% CO2. After *in vitro* stimulation, supernatants were collected and stored in −20°C until used for ELISA. Whole blood stimulation experiments were conducted using 100μl of heparin blood that was added to a 48 well plate and subsequently stimulated with 400μl of LPS, PHA (final volume 500ul) and *Staphylococcus Aureus* for 48 hours at 37°C and 5% CO2. Supernatants were collected and stored in -20°C until used for ELISA. Concentrations of human TNFα, IFNγ, IL1Ra, IL1β, IL6, IL8, IL10, IL17, and IL22 were determined using specific commercial ELISA kits (PeliKine Compact, Amsterdam, or R&D Systems), in accordance with the manufacturer’s instructions. When values were below or above the detection limit of the ELISA, the corresponding limit was used. After log transformation of cytokine quantitative trait loci (cQTL) data, linear regression analyses adjusted for age and sex were used to determine the correlation of the SNPs with cQTLs. All analyses were performed using R software (<http://www.r-project.org/>) using custom scripts in the R programming language based on existing functions such as lm (stats). In order to account for multiple comparisons, we used a significance threshold of 2.26•10^-5^ (0.05/(41 independent SNPs x 9 cytokines x 6 stimulants)).

*Correlation of GWAS-identified SNPs and blood cell counts and blood proteomic profile*

We also investigated the effect of the GWAS variants on cell-level variation by using a set of 91 manually annotated immune cell populations and genotype data from the HFGP cohort that included 408 healthy subjects (Supplementary table 4). Cell populations were measured by 10-color flow cytometry (Navios flow cytometer, Beckman Coulter) after blood sampling (2-3 hours) and cell count analysis was performed using the Kaluza software (Beckman Coulter, v.1.3). In order to reduce inter-experimental noise and increase statistical power cell count analysis was performed by calculating parental and grandparental percentages, which were defined as the percentage of a certain cell type within the subpopulation of cells from which it was isolated^7^. Detailed laboratory protocols for cell isolation, reagents, gating and flow cytometry analysis have been reported elsewhere^8^ and the accession number for the raw flow cytometry data and analysed data files are available upon request to the authors (<http://hfgp.bbmri.nl>). A proteomic analysis was also performed in serum and plasma samples from the HFGP cohort. Circulating proteins were measured using the commercial Olink® Inflammation panel (Olink, Sweden) that resulted in the measurement of 103 different biomarkers (Supplementary table 5). Protein levels were expressed on a log2-scale as normalized protein expression values, and normalized using bridging samples to correct for batch variation. Considering the number of proteins (n=103), blood-derived cell populations (n=91), and SNPs (n=41) tested, significance P-values were set to be 1.18•10^-5^ and 1.34•10^-5^ for the proteomic and blood cell count analyses, respectively.

*Correlation between GWAS-identified SNPs and plasma steroid hormone concentrations*

Next, we investigated the correlation of the SNPs with levels of 7 plasma steroid hormones (androstenedione, cortisol, 11-deoxy-cortisol, 17-hydroxy progesterone, progesterone, testosterone and 25 hydroxy vitamin D3) in 279 subjects selected from the HFGP project that did not have hormone replacement therapies or used oral contraceptives. Plasma steroid hormone concentrations were determined by chromatography-tandem mass spectrometry after protein precipitation and solid-phase extraction following previously reported protocols^8^. After log transformation, correlation between steroid hormone levels and GWAS-identified SNPs was evaluated by linear regression analysis adjusted for age and sex. Significance threshold was set to 0.000174 considering the number of independent SNPs tested (n=41) and the number of hormones determined (n=7).

**Supplementary Table 4.** Cell types analysed either in whole blood or peripheral mononuclear blood cells.

**Supplementary table 5.** Serum and plasma metabolites measured in the HFGP cohort.

*Predictive models and discriminative accuracy using AUROC and PRS approaches.*

The value of validated GWAS variants for prediction of CLL onset was assessed using stepwise logistic regression. Models were built including demographic variables (age, sex and country of origin) and genetic polymorphisms that showed significant associations with CLL risk in the CRuCIAL population (*P*<0.05) and after the meta-analysis of the CRuCIAL data with those from previously published GWAS (*P*<5.0•10^-8^). The genetic model was then compared with the reference model including demographic variables. The area under the curve (AUC) of a receiver operating characteristic (ROC) curve analysis and −2 log likelihood ratio (LR) tests were used to assess whether the genetic models fitted significantly better the data compared to their respective reference models. Finally, we run randomization tests to confirm whether the improved predictive ability of each genetic/functional model was consistent after 50.000 iterations. All tests were conducted using R software (<http://www.r-project.org/>; Supplementary table 6).

**Supplementary table 6**. Discriminative value *AUC* for models including GWAS-identified variants for CLL.

| **Reference model*** | | | | | | **LR test**  ***P*-value** |
| --- | --- | --- | --- | --- | --- | --- |
|  |  | | P-value | OR 95%CI | AUC 95%CI |  |
| Age |  | | 2.00E-16 | 1.088 (1.078-1.098) |  |  |
| Gender |  | | 1.55E-10 | 0.519 (0.425-0.635) | 0.765 (0.744-0.787)^∂^ |  |
| **Predictive model including 16 SNPs**** | | | | | |  |
|  | |  | P-value | OR 95%CI | AUC 95%CI^a^ |  |
| *ODF3B*_rs140522_Rec | |  | 0.082 | 1.343 (0.963-1.875) |  |  |
| *IRF8*_rs391855_Dom | |  | 0.058 | 1.309 (0.991-1.730) |  |  |
| *QPCT\|RNU6-1116P*_rs888096_Rec | |  | 0.031 | 1.376 (1.028-1.842) |  |  |
| *DMRTA1*_rs1359742_Dom | |  | 0.010 | 1.362 (1.075-1.726) |  |  |
| *ACOXL*_rs1439287_Rec | |  | 0.00016 | 1.618 (1.260-2.078) |  |  |
| *OPRM1\|\|IPCEF1*_rs2236256_Dom | |  | 0.091 | 1.211 (0.970-1.512) |  |  |
| *AC107990.1\|\|NFE2L3P1*_rs4368253_Rec | |  | 0.018 | 1.284 (1.043-1.580) |  |  |
| *BCL2*_rs4987855_Rec | |  | 0.007 | 1.478 (1.110-1.968) |  |  |
| *FAS*_rs6586163_Rec | |  | 7.35•10^-5^ | 1.607 (1.271-2.033) |  |  |
| *IRF4*_rs9392504_Rec | |  | 7.24•10^-9^ | 1.988 (1.575-2.509) |  |  |
| *EOMES\|LINC01980*_rs9880772_Rec | |  | 0.004 | 1.448 (1.122-1.869) |  |  |
| *MYNN*_rs10936599_Rec | |  | 0.095 | 1.202 (0.968-1.491) |  |  |
| *PRKD2*_rs11083846_Rec | |  | 0.027 | 1.580 (1.052-2.373) |  |  |
| *PCAT29\|LOC107984788*_rs11637565_Rec | |  | 0.0007 | 1.580 (1.211-2.061) |  |  |
| *POT1*_rs17246404_dom | |  | 0.0036 | 1.847 (1.222-2.791) |  |  |
| *GRAMD1B*_rs35923643_Dom | |  | 9.22•10^-14^ | 2.238 (1.811-2.766) |  |  |
| Age | |  | 2.00E-16 | 1.091 (1.080-1.101) |  |  |
| Gender | |  | 2.69E-10 | 0.505 (0.409-0.624) | 0.809 (0.790-0.828)^∂^ | **2.2•10^-16^** |

* Including age and gender as variable never dropped from models and compared with a baseline model with AUROC=0.5.

**SNPs showing a significant association with CLL risk (*P*<0.10).

After removing missing values, 2122 subjects were available for prediction capacity analysis.

∂ A LR test showed that the model including genetic variants fitted better the data than the reference model and that the difference in model fit between both models was statistically significant (-2log likehood ratio test, df=-16, ***P*=2.2E-16**).

Residual deviance (Reference model): 2375.5

Null deviance (Reference model: 2793.2

Residual deviance (Genetic model): 2178.1

Null deviance (Genetic model: 2793.2

Given the noticeable impact of demographic variables in predicting disease risk, we also computed weighted and unweighted polygenic risk score (PRS) that could provide additional information about the strength of genetic variants to predict disease risk. For that purpose, the number of alleles associated with higher CLL risk were counted and added up for each study subject, resulting in an unweighted PRS, which had a theoretical range from 0 (no CLL risk alleles) to 16 (all risk alleles are present at each SNP in homozygosity). In addition, we built a weighted PRS by using the ORs of the codominant model of the association of each variant with CLL risk in the CRuCIAL population as coefficients to weight the relative effects of the risk SNPs. For each SNP in the weighted PRS, a value of 0 was assigned if 0 risk alleles were present and the ln(OR) of the heterozygous or homozygous was respectively assigned if one or two risk alleles were present. Then all the values were summed among them for each subject. We built an alternative weighted PRS by using ORs from previously published GWAS (Supplementary Table 7). For both PRSs (weighted and unweighted), we calculated quintiles based on the distribution of values in the controls. The formulas for the unweighted and weighted scores are respectively Σm1 aj and Σm1 aXj, where a = number of risk alleles (0, 1, 2), m = total number of SNPs (16), j = jth subject, X =ln(OR). A detailed explanation of how the scores were generated has been previously reported^9^.

**Supplementary Table 7**. Associations between PRSs and CLL risk with the different types of scores

| **Type of polygenic risk score** | | | | **AUROC** |
| --- | --- | --- | --- | --- |
|  | Quintiles | OR 95%CI^a^ | P-value | AUROC (95%CI) |
| Unweighted, subjects with 100% call rate | 1 | 1.00 |  |  |
|  | 2 | 1.87 (1.29-2.71) | 1.04•10^-03^ |  |
|  | 3 | 3.05 (2.05-4.54) | 4.00•10^-08^ |  |
|  | 4 | 3.78 (2.64-5.43) | 5.30•10^-13^ |  |
|  | 5 | 6.81 (4.65-9.96) | 2.00•10^-21^ |  |
|  | Continuos^b^ | 1.55 (1.44-1.67) | 5.38•10^-12^ | 0.65 (0.63-0.67) |
|  |  |  |  |  |
| Weighted, subjects with 100% call rate | 1 | 1.00 |  |  |
|  | 2 | 2.91 (1.87-4.53) | 2.32•10^-06^ |  |
|  | 3 | 3.50 (2.28-5.38) | 1.19•10^-08^ |  |
|  | 4 | 5.43 (3.58-8.23) | 2.00•10^-15^ |  |
|  | 5 | 10.45 (6.96-15.7) | 2.00•10^-27^ |  |
|  | Continuos^b^ | 1.67 (1.54-1.80) | 4.08•10^-12^ | 0.68 (0.65-0.70) |
|  |  |  |  |  |
| Weighted 100% call rate using GWAS OR^c^ | 1 | 1.00 |  |  |
|  | 2 | 1.83 (1.20-2.80) | 4.94•10^-03^ |  |
|  | 3 | 3.26 (2.19-4.87) | 7.22•10^-09^ |  |
|  | 4 | 3.85 (2.60-5.71) | 1.82•10^-11^ |  |
|  | 5 | 7.68 (5.25-11.2) | 4.00•10^-24^ |  |
|  | Continuos^b^ | 1.61 (1.49-1.74) | 2.06•10^-11^ | 0.66 (0.64-0.69) |

^a^ OR, odds ratio; CI, confidence interval. All analyses were adjusted for age, sex and geographic region of origin.

^b^ The unit for the analysis with the continuous variable was the increment of one quintile.

^c^ The weights used to build this score were the ORs of the associations between the individual SNPs and CLL risk observed in previous GWAS studies.

**Supplementary Figure 1.** Correlation between *ILRUN* and *POU5F1P2|ODF1* SNPs and blood-derived cell populations. **
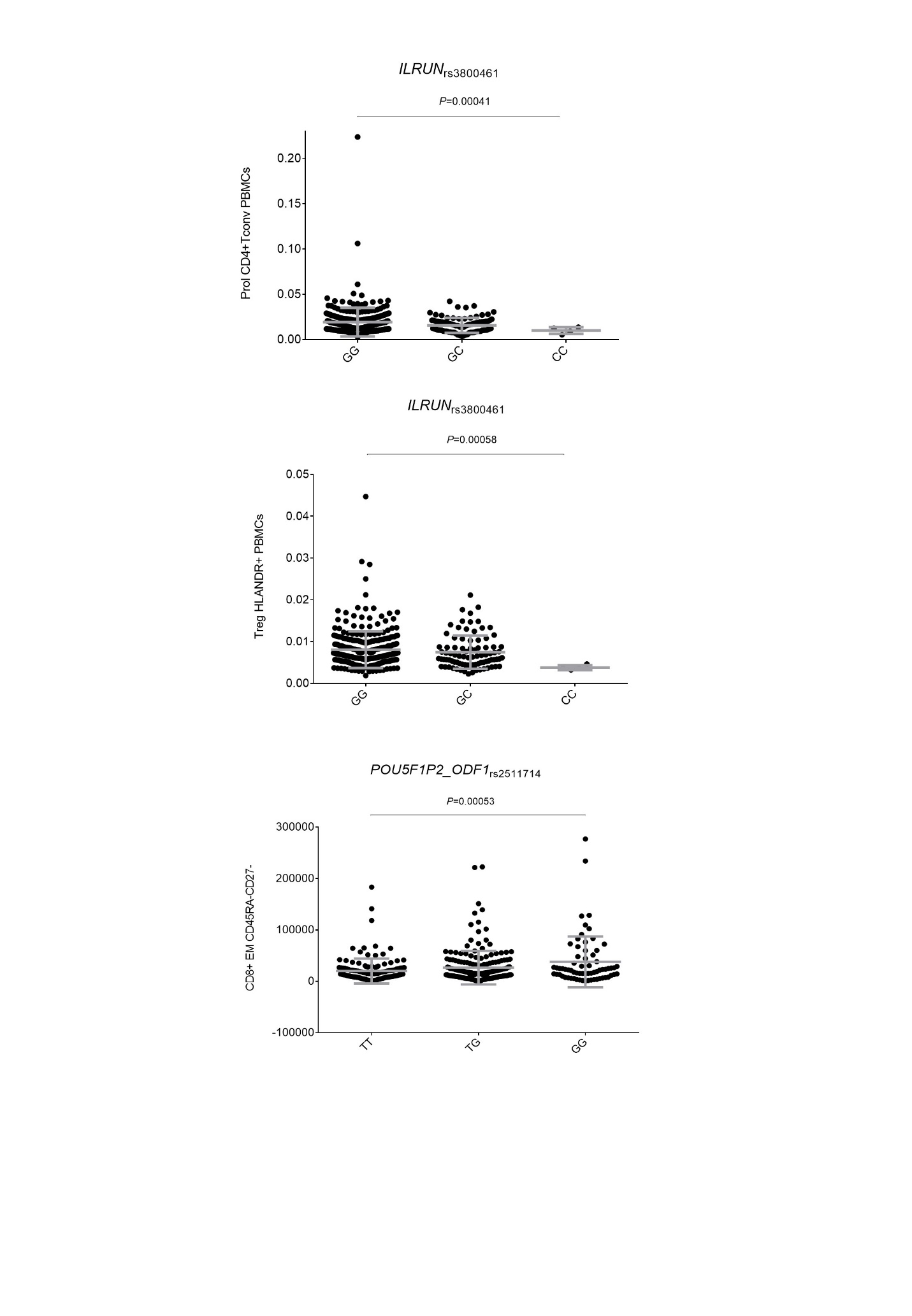
**

**REFERENCES**

1 Hallek, M. *et al.* Guidelines for the diagnosis and treatment of chronic lymphocytic leukemia: a report from the International Workshop on Chronic Lymphocytic Leukemia updating the National Cancer Institute-Working Group 1996 guidelines. *Blood* **111**, 5446-5456, doi:10.1182/blood-2007-06-093906 (2008).

2 Hallek, M. *et al.* iwCLL guidelines for diagnosis, indications for treatment, response assessment, and supportive management of CLL. *Blood* **131**, 2745-2760, doi:10.1182/blood-2017-09-806398 (2018).

3 Berndt, S. I. *et al.* Meta-analysis of genome-wide association studies discovers multiple loci for chronic lymphocytic leukemia. *Nat Commun* **7**, 10933, doi:10.1038/ncomms10933 (2016).

4 Speedy, H. E. *et al.* A genome-wide association study identifies multiple susceptibility loci for chronic lymphocytic leukemia. *Nat Genet* **46**, 56-60, doi:10.1038/ng.2843 (2014).

5 Berndt, S. I. *et al.* Genome-wide association study identifies multiple risk loci for chronic lymphocytic leukemia. *Nat Genet* **45**, 868-876, doi:10.1038/ng.2652 (2013).

6 Slager, S. L. *et al.* Common variation at 6p21.31 (BAK1) influences the risk of chronic lymphocytic leukemia. *Blood* **120**, 843-846, doi:10.1182/blood-2012-03-413591 (2012).

7 Orru, V. *et al.* Genetic variants regulating immune cell levels in health and disease. *Cell* **155**, 242-256, doi:10.1016/j.cell.2013.08.041 (2013).

8 Aguirre-Gamboa, R. *et al.* Differential Effects of Environmental and Genetic Factors on T and B Cell Immune Traits. *Cell Rep* **17**, 2474-2487, doi:10.1016/j.celrep.2016.10.053 (2016).

9 Canzian, F. *et al.* A polygenic risk score for multiple myeloma risk prediction. *Eur J Hum Genet*, doi:10.1038/s41431-021-00986-8 (2021).
